# The HMGB Protein *Kl*Ixr1, a DNA Binding Regulator of *Kluyveromyces lactis* Gene Expression Involved in Oxidative Metabolism, Growth, and dNTP Synthesis

**DOI:** 10.3390/biom11091392

**Published:** 2021-09-21

**Authors:** Agustín Rico-Díaz, Aída Barreiro-Alonso, Cora Rey-Souto, Manuel Becerra, Mónica Lamas-Maceiras, M. Esperanza Cerdán, Ángel Vizoso-Vázquez

**Affiliations:** Grupo EXPRELA, Centro de Investigacións Científicas Avanzadas (CICA), Departamento de Bioloxía, Facultade de Ciencias, INIBIC—Universidade de A Coruña, Campus de A Zapateira, 15071 A Coruña, Spain; aricodiaz@gmail.com (A.R.-D.); aida.barreiro@udc.es (A.B.-A.); cora.rey.souto@udc.es (C.R.-S.); manuel.becerra@udc.es (M.B.); monica.lamas@udc.es (M.L.-M.); esper.cerdan@udc.es (M.E.C.)

**Keywords:** *Kluyveromyces lactis*, IXR1, cisplatin sensitivity, hypoxia, dNTP pool

## Abstract

In the traditional fermentative model yeast *Saccharomyces cerevisiae*, *Sc*Ixr1 is an HMGB (High Mobility Group box B) protein that has been considered as an important regulator of gene transcription in response to external changes like oxygen, carbon source, or nutrient availability. *Kluyveromyces lactis* is also a useful eukaryotic model, more similar to many human cells due to its respiratory metabolism. We cloned and functionally characterized by different methodologies *Kl*IXR1, which encodes a protein with only 34.4% amino acid sequence similarity to *Sc*Ixr1. Our data indicate that both proteins share common functions, including their involvement in the response to hypoxia or oxidative stress induced by hydrogen peroxide or metal treatments, as well as in the control of key regulators for maintenance of the dNTP (deoxyribonucleotide triphosphate) pool and ribosome synthesis. *Kl*Ixr1 is able to bind specific regulatory DNA sequences in the promoter of its target genes, which are well conserved between *S. cerevisiae* and *K. lactis*. Oppositely, we found important differences between *Sc*Irx1 and *Kl*Ixr1 affecting cellular responses to cisplatin or cycloheximide in these yeasts, which could be dependent on specific and non-conserved domains present in these two proteins.

## 1. Introduction

Easy manipulation and availability of high throughput platforms to evaluate the response of yeast cells to directed mutations and drugs strengthen the rationale of using these organisms as disease models, or for testing chemical libraries to find new therapeutic treatments [1,2,3]. *Saccharomyces cerevisiae*, *Candida albicans,* or *Schizosaccharomyces pombe* [4] have been traditionally used with this purpose or as cell factories, but there are many other yeasts that could be also included as far as the knowledge of their biology and regulatory molecular mechanisms controlling principal functions would increase. *Kluyveromyces lactis* is a good candidate that has been extensively studied in the last twenty years as a non-conventional yeast [5]. 

The HMGB protein family is characterized for presenting one or more HMG-box domains with DNA/RNA binding abilities. In multicellular eukaryotes, these proteins are implicated not only in many DNA-dependent nuclear processes at the chromatin level [6] (sequence and non-sequence specific), but also in the cytoplasm [7], or extracellularly acting in cell signaling and inflammation [8]. In the nucleus HMGB proteins from yeast and other higher eukaryotes are functionally involved in chromatin interactions, DNA repair, transcriptional regulation, and epigenetic control of gene expression. Cytoplasmic functions of HMGB proteins, like human HMGB1, are related to the balance between apoptosis and autophagy [9]. The binding of HMGB1 to beclin1 promotes autophagy and simultaneously inhibits apoptosis [10], meanwhile the interaction of HMGB1 with p53 diminishes HMGB1-beclin1 interactions in the cytoplasm and promotes apoptosis [11]. Besides, HMGB proteins may function as alarmins and coordinate multicellular responses during immune response and inflammation [12]. Interestingly, HMGB proteins are also associated with diverse diseases like cancer [13], kidney disease [14], rheumatoid arthritis [15], Alzheimer’s disease [16], among many others [17]. HMGB proteins are well characterized in *S. cerevisiae*, a yeast with a predominant fermentative metabolism [18], but not in other yeasts, like *K. lactis*, envisaged as an advantageous eukaryote model with respiratory metabolism [19,20]. It has been proposed that during aerobic growth, the high oxidative metabolism observed in *K. lactis* depends on the pentose phosphate pathway (PPP) and on a very active mitochondrial respiration [20].

In *S. cerevisiae,* seven HMGB proteins have been characterized, *Sc*Abf2, *Sc*Hmo1, *Sc*Ixr1, *Sc*Nhp6A, *Sc*Nhp6B, *Sc*Nhp10, and *Sc*Rox1, but their homologs in other yeast are less known. *Sc*Rox1 (regulation by oxygen 1) and *Sc*Ixr1 (intrastrand cross (X)-link recognition 1) proteins take part in the regulation of *ScHEM13* (HEMe biosynthesis 13) and other hypoxic genes [21,22,23,24,25,26]. In aerobiosis, *Sc*Rox1, in cooperation with *Sc*Mot3 (modifier of transcription 3), represses the expression of *ScHEM13* [14,27] and also represses *ScIXR1* expression [23]. During hypoxia, *ScROX1* is downregulated, *ScIXR1* expression rises, and *Sc*Ixr1 activates *ScHEM13* transcription [23]. *Sc*Ixr1 also participates in the oxidative response caused by hydrogen peroxide in *S. cerevisiae* [23]. Besides, the protein binds to the adducts that cisplatin forms with DNA [27] and its depletion causes increased resistance to cisplatin [28]. The transcriptional regulation on ribosomal RNAs and genes encoding ribosomal proteins or involved in ribosome assembly exerted by *Sc*Ixr1 is directly related to *S. cerevisiae* response to cisplatin treatment [29,30]. *Sc*Ixr1 is also required for the maintenance of an adequate supply and balance of dNTPs for DNA synthesis [31,32] and repair [33], exerting a regulatory control of *ScRNR1* (ribonucleotide reductase 1) expression and participating in the genome integrity checkpoint signaling pathway leaded by the kinases *Sc*Mec1 (mitosis entry checkpoint 1) and *Sc*Rad53 (radiation sensitive 53).

Considering *K. lactis* as an alternative model to *S. cerevisiae*, showing a predominant respiratory metabolism, functional characterization of HMGB proteins in this yeast and comparative analyses in reference to those of *S. cerevisiae* is a pending task that may report important insights about their role as master regulators in response to changes in environmental conditions, which have huge regulatory influences in unicellular eukaryotes [20,34]. In *K. lactis*, transcriptional factors are among those proteins showing the lowest similitude to their *S. cerevisiae* counterparts, and besides, functional homology is also limited [19,20,21]. This rule is applicable to HMGB proteins. *Kl*Rox1, the only HMGB protein studied in *K. lactis* so far [35], does not regulate the hypoxic response [36,37], but participates in the response to cadmium and arsenate by regulating the *KlYCF1* promoter [35]. In this work, we cloned and functionally characterized *KlIXR1*, which encodes a protein with only 34.4% sequence similarity to *Sc*Ixr1. Our data indicate that both proteins share common functions, including their involvement in the response to hypoxia or induced oxidative stress, as well as in the control of key regulators for maintenance of the dNTP pool and ribosome synthesis. Nevertheless, we found differences between *Sc*Ixr1 and *Kl*Ixr1 affecting cellular responses to cisplatin or cycloheximide. These characteristics might be considered when using *K. lactis* as a eukaryote model for high throughput screenings.

## 2. Materials and Methods

### 2.1. Yeast Strains, Media, and Growth Condition

The *S. cerevisiae* strain W303 and its isogenic derivative W303-*ixr1Δ* have been previously described [37]. The *K. lactis* strain MW190-9B (MAT*a*, *lac4-8*, *uraA*, Rag+) was kindly provided by M. Wesolowski-Louvel (Université Claude Bernard, France).

Cells were grown at 30 °C in YPD (2% glucose (*w/v*), 2% bacto-peptone (*w/v*), 1% yeast extract (*w/v*)), or complete media, CM, prepared as reported [38] with different carbon sources or specific nutrient omissions as specified. Media for hypoxic growth were supplemented with 0.002% ergosterol in ethanol (*w/v*) and 0.5% Tween 80 (*v/v*). Ergosterol biosynthesis in yeast is limited in hypoxic conditions by defects in specific enzymes that depend on oxygen and heme as an essential substrate or cofactor, meanwhile surfactant Tween 80 maintains ergosterol soluble in polar solvent and is available to be taken up by the cell. The AnaeroGen system (Oxoid Ltd., Dublin, Ireland) was used for hypoxic growth, and oxygen levels were monitored by the BR55 indicator strip (Oxoid Ltd., Dublin, Ireland). For analysis of resistance to metals, cisplatin was added to the media in the form of cis-dichlorodiammineplatinum (II) (Sigma Aldrich Chemicals Co., St. Louis, MO, USA), and Cd (II) was added to the media in the form of cadmium sulphate 8/3-hydrate (Sigma Aldrich Chemicals Co., St. Louis, MO, USA). All concentrations are specified in the text and figures. For analysis of the *petite* phenotype, hemin (Sigma Aldrich Chemicals Co., St. Louis, MO, USA) was added at a final concentration of 50 µg/mL and S-adenosyl homocysteine (Sigma Aldrich Chemicals Co., St. Louis, MO, USA) at a final concentration of 1 mM. 

### 2.2. General Techniques

DNA isolation, propagation, and modification were carried out using standard techniques as previously described [39]. Yeast genomic sequences were obtained from the *Saccharomyces* Genome Database (SGD) maintained at Stanford University (http://www.yeastgenome.org; last accessed date: 1 April 2021) or from Ensembl (https://fungi.ensembl.org/index.html; last accessed date: 1 April 2021) databases. In silico analyses of promoters were done with RSA (Regulatory Sequence Analysis) tools (http://rsat.sb-roscoff.fr; last accessed date: 11 April 2021) and YEASTRACT (Yeast Search for Transcriptional Regulators and Consensus Tracking) repository (http://www.yeastract.com; last accessed date: 11 April 2021), using the consensus sequences YYYATTGTTCTC and KTTSAAYKGTTYASA, previously described for *Sc*Ixr1 binding in *S. cerevisiae* [23,24]. The primers used in this work are described in Supplementary Table S1.

The clone of *KlIXR1* in the shuttle vector YEplac195 [40] was obtained by PCR amplification of genomic DNA from the *K. lactis* strain YRRL-Y1140 with the primers ECV719 and ECV720 (Table S1). The PCR product, containing the *KlIXR1* ORF (Open Reading Frame), a 698 bp upstream region, and a 643 bp downstream region, was digested with the enzymes *SphI* and *SalI* (included in the PCR primers) and introduced in the same sites of the MCS (Multi Cloning Site) of the plasmid. 

The clone of *KlIXR1* ORF in the vector pAG426GAL-ccdB-EGFP (Addgene plasmid #14203) was obtained by PCR amplification of genomic DNA from the *K. lactis* strain YRRL-Y1140 with the primers AJVV024f and AJVV025r (described in Table S1). A 6xHis sequence was included in AJVV024f primer for N-terminus tagging of protein product. 

Both clones, YEplac195-*KlIXR1* and pAG426GAL-*KlIXR1*, were verified by restriction analysis and sequenced.

### 2.3. Construction and Verification of the KlIXR1 Null Strain

The construction YEplac195-*KlIXR1* was used as template for inverse PCR with divergent primers, ECV763 and ECV764, carrying *NotI* restriction sites at the 5’ ends, and producing an internal deletion between positions +118 and +1075 of the *KlIXR1* coding sequence. The *kanMX4* cassette obtained from the pFA6-*kanMX4* plasmid [41] was then introduced between *NotI* sites by T4 ligation. This new construction, YEplac195-*KlIXR1*::*kanMX4*, was used as template to amplify by PCR using the ECV765 and ECV766 primers. The resulted linear fragment contained the marker for geneticin (G418) selection and two flanking regions of homology to the *K. lactis* genome, extended to 730 bp in the 5′ region up the *Kl*Ixr1 deletion and to 664 bp in the 3′ region. Cells of the *K. lactis* strain MW190-9B transformed with this fragment were selected in CM plates supplemented with 200 µg/mL geneticin. To verify, the correct replacement in the *K. lactis* genome was verified by PCR as previously described [42]. Genomic DNAs isolated from the MW190-9B strain (wild type) and the null candidates were amplified by PCR combining one internal primer designed for annealing inside *kanMX4* (ECV315K2 and ECV314K3) with one external primer designed for annealing in the genome flanking to *KlIXR1* gene, but external to the regions of homology used for the recombination event (ECV808AR and ECV809AR, respectively). The correct size of the expected amplicons was verified (Supplementary Figure S1).

### 2.4. Northern Blotting

RNA was isolated from *S. cerevisiae* or *K. lactis* yeast cells grown in different media to A_600_ = 0.8, and analyzed as described previously [39] with minor modifications. PCR amplification with specific primers were used to obtain DNA probes for each gene (Table S1). For each condition, three independent Northern blot experiments were performed to ensure reliable results. Data from all the analysis were included in the quantification, but figures only show one representative experiment. Results were normalized for RNA loading against the signal obtained from the control probe, *KlSNR17A*. The value 1 in arbitrary units (A.U.) was designed to control experiment and used as the reference for re-scaling the other signals. 

### 2.5. Analysis of Expression by RT-qPCR 

Total RNA isolated as previously described was converted into cDNA and labeled with the One-step NZYSpeedy RT-qPCR Green kit (NZYTech, Lisboa, Portugal). PCR primers were designed following procedures previously described [43]. The sequence of primers is available in Supplementary Material online resources accompanying this paper (Supplementary Table S1). The StepOnePlus Real-Time PCR System was used for the experiments (Applied Biosystems, Austin, TX, USA) and calculations were made by the 2^−ΔΔCt^ method [44]. Three independent RNA extractions and two technical replicates were assayed for each strain or condition. The mRNA levels of the selected genes were corrected by the geometric mean of the mRNA levels of *KlTAF10*, previously verified to be constitutive in the assayed conditions and not affected by the deletion. A paired Student’s *t*-test with two-tails was applied to evaluate the differences between Δ*C*t values (Ct values normalized with reference gene) of control and treated samples. A *p*-value lower than 0.05 was considered significant, although results with a significance lower of 0.01 were also indicated.

### 2.6. Isolation of KlIxr1 Protein

Protein samples used in the EMSA assays were obtained by expressing full-length *Kl*Ixr1, fused at its N-terminus to a 6xHis tag, in *E. coli* BL21-DE3 cells (Sigma Aldrich Chemicals Co., St. Louis, MO, USA). The ORF was amplified from the YEplac195-*KlIXR1* construction by PCR, using the primers AVV021 and AVV022 (Table S1). The DNA insert, previously digested with the enzymes *VspI* and *HindIII,* was ligated into the expression vector pOPTH ([ampr ori 2μm His6]) digested with the enzymes *NdeI* and *HindIII*. The new construction, pOPTH-*KlIXR1*, was verified by restriction analysis and sequencing. Competent *E. coli* BL21-DE3 cells were transformed with pOPTH-*KlIXR1* and grown in LBA at 37 °C to reach OD_600_ = 1.0. Then, expression was induced with 0.1 mM IPTG (isopropyl-alfa-D-thiogalactopyranoside, Sigma Aldrich Chemicals Co., St. Louis, MO, USA), and growth was continued during 3 h at 37 °C. After expression, cell pellets were collected and lysed by sonication in high salt lysis buffer (50 mM Tris-HCl buffer pH 8.0, 1 M NaCl, 2 mM dithiothreitol and 2× Complete Mini EDTA-free protease inhibitor cocktail from Roche, Switzerland). After clarification by centrifugation 30 min at 23,000× *g*, lysates were passed through HisTrap HP Column, 5 mL (GE Healthcare, Chicago, IL, EEUU) equilibrated in wash buffer A (50 mM Tris-HCl buffer pH 8.0, 100 mM NaCl, 2 mM dithiothreitol). Proteins were eluted in an AKTA prime plus (GE Healthcare, EEUU) by linear gradient from 0 to 100% of buffer elution B (50 mM Tris-HCl buffer pH 8.0, 100 mM NaCl, 2 mM dithiothreitol, 300 mM imidazole). After elution, protein was further purified by gel filtration chromatography using a HiLoad™ 16/600 Superdex™ 200 pg column (GE Healthcare, Chicago, IL, EEUU) pre-equilibrated with SEC buffer (20 mM Tris-HCl buffer pH 8.0, 100 mM NaCl, 1 mM dithiothreitol, 1 mM EDTA). The protein was concentrated by ultrafiltration using Amicon Ultra 15 mL Centrifugal Filters, 10 kDa (Merck, Darmstadt, Germany). Final homogeneity of *Kl*Ixr1 protein was verified by SDS-PAGE (Supplementary Figure S2), observing a protein with the expected size (51.3 kDa).

### 2.7. Electrophoretic Mobility Shift Assay

EMSA (Electrophoretic Mobility Shift Assay) assays were carried out to test *Kl*Ixr1 binding to the *KlHEM13* and *KlYCF1* promoters. The desired promoter target was obtained after annealing complementary primers described in Supplementary Table S1, by mixing the complementary oligonucleotides in equimolar amounts, heating to 95 °C for 5 min, and cooling slowly to room temperature in darkness. The 5′-ends of forward primers were fluorescein-labeled (FAM) for fluorescence detection. The binding reactions (20 µL) were performed at 4 °C for 30 min in the buffer previously described [21] with 500 nM DNA 5′ labeled, 1 μg of the assayed protein, and 1 μg of calf thymus DNA (Sigma-Aldrich, USA) as carrier. In competition experiments, an excess (1-, 2-, 5- or 100-fold molar) of the same or mutated versions of unlabeled promoter fragment were used as a specific competitor. A 100-fold molar excess of non-labeled fragmented salmon sperm DNA (Sigma Aldrich Chemicals Co., St. Louis, MO, USA) was used as the nonspecific competitor (data not shown). Samples were electrophoresed as previously described [29] and the gels scanned for fluorescence in a Typhoon FLA 7000 Biomolecular Imager v.1.2 (GE Healthcare, Chicago, IL, USA) to detect the FAM fluorophore, using 473 nm laser excitation and an Y520 filter.

### 2.8. Fluorescence Anisotropy Experiments

FAM-labeled dsDNA oligonucleotides (see Supplementary Table S1) were extensively dialyzed against the buffer (10 mM Tris-HCl pH 8.0, 100 mM KCl, 2 mM DTT, 1 mM EDTA, 500 μg/mL bovine serum albumin). Fluorescence anisotropy (FA) titrations were performed at 25 °C on a Multi-modal Synergy H1 plate reader (Biotek, Winooski, VT, USA) using 384 Low Volume Black Round Bottom Polystyrene NBS Microplate (Corning, New York, NY, USA) with 15 μL per well. The excitation and detection wavelengths were 485 and 528 nm, respectively, with dichroic mirror (510 nm) and polarizer filter assembled. Tumbling rates or changes in the rotational times of the small labelled-DNAs, when tightly bound to large proteins, were used to calculate fluorescence anisotropy values. In each titration, the fluorescence anisotropy of a solution of 100 nM FAM-tagged duplex DNA was measured, normalized, and represented in a percentage of ligand bound as a function of the added protein concentration. For each competition experiment, the polarization signal was followed over time. As a result, a 30 min incubation period was selected as an adequate time to reach equilibrium (data not shown). Binding data were fitted to a simple one site saturation-binding model by nonlinear least squares regression using GraphPad Prism 6.0 (GraphPad Software Inc., San Diego, CA, USA). Each titration was performed three times, and the final affinity was taken as the mean of these measurements. In competition experiments, IC50 values were determined using GraphPad Prism 6.0.

### 2.9. Bioinformatics Analysis and 3-D Modelling

Alignment was done by the local alignment tool LALIGN (https://www.ebi.ac.uk/Tools/psa/lalign/; last accessed date: 5 February 2021) using the BLOSUM50 matrix and edited by ESPript 3 (http://espript.ibcp.fr/ESPript/ESPript/index.php; last accessed date: 5 February 2021). 3-D Homology modelling of *Kl*Ixr1 and *Sc*Ixr1 HMG-box domains was done by Phyre2 server [45] (http://www.sbg.bio.ic.ac.uk/phyre2/html/page.cgi?id=index; last accessed date: 6 May 2021), based on protein templates human HMGB1 (PDB *2E6O*), human SOX9 (PDB *4EUW*), human SOX17 (PDB *2YUL*), and Tox2 protein from *Mus musculus* (PDB *2CO9*) in the Protein Data Bank (http://www.rcsb.org; last accessed date: 6 May 2021). Protein model pictures were made with the PyMol package v1.7 (www.pymol.org, 1st February 2014) and structure similarities were analyzed with the TM-align tool [46] (https://zhanggroup.org/TM-align/; last accessed date: 15 May 2021).

## 3. Results

### 3.1. Heterologous Expression of KLLA0E18481g (KlIXR1) in Saccharomyces Cerevisiae Does Not Complement the Increased Resistance to Cisplatin Observed in the S. cerevisiae ixr1Δ Mutant

A pairwise local alignment of the amino acid sequences of the ORFs *YKL032c*, the *Sc*Ixr1 protein of *S. cerevisiae*, and *KLLA0E18481g* (*Kl*Ixr1) from *K. lactis* reveals only 34.4% amino acid sequence similarity (considered as substitutions for amino acids of the same chemical group) and 26.7% identity between these two proteins. 

**Figure 1:**
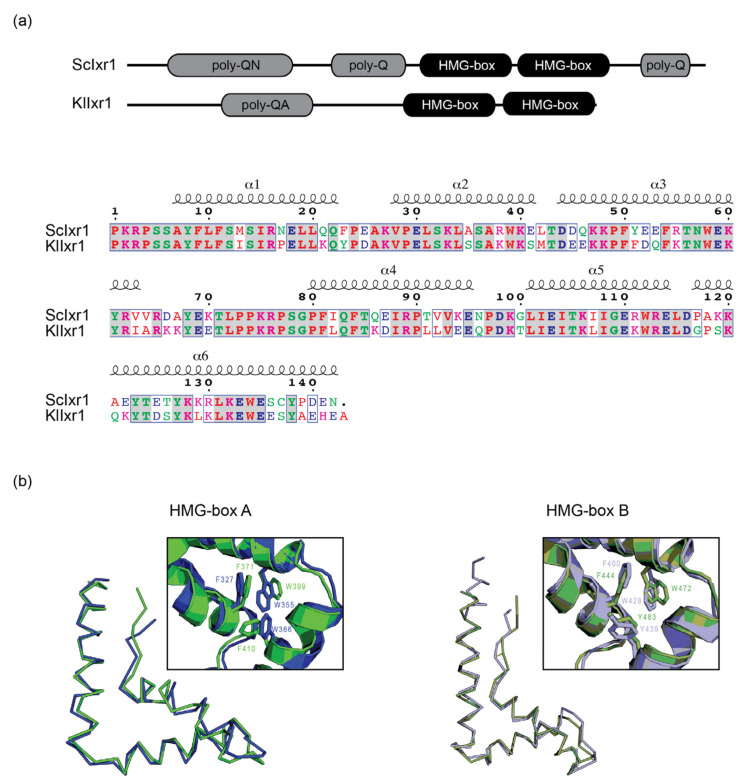
(**a**) In silico comparison of Ixr1 amino acid sequence from *S. cerevisiae* and *K. lactis*.(upper) Scheme of protein domain distribution in Ixr1 protein from *S. cerevisiae* and *K. lactis.* (lower) Pairwise alignment of the Ixr1 double HMG-box domains arranged in tandem from *S. cerevisiae* (Uniprot code *P33417*) and *K. lactis* (Uniprot code *Q6CMQ4*). Conserved amino acids are highlighted in bold and conserved regions indicated by grey boxes. Small and hydrophobic amino acids (less tyrosine) are indicated in red, acidic amino acids in blue, basic amino acids (less histidine) in magenta and hydroxyl + sulfhydryl + amine (STYHCNGQ) in green. (**b**) Model superposition of HMG-box A (*S. cerevisiae* in dark green and *K. lactis* in dark blue) and HMG-box B (*S. cerevisiae* in light green and *K. lactis* in light blue). Enlarged frames show the conserved aromatic residues that form the cluster stabilizing the three-helix HMG folding.

In fact, the region actually conserved extends only to the sequence that includes the two HMG-box domains present in both proteins. The long traits of poly-glutamines and asparagines found in *Sc*Ixr1 do not exist in *Kl*Ixr1 and a region with repeated glutamines and alanines present in *Kl*Ixr1 is absent in *Sc*Ixr1 (Figure 1a). The conserved amino acid sequences in the HMG domains have been aligned with the program LALIGN showing a 91.3% similarity and 70.3% identity between both HMG-box tandem regions (Figure 1a). Additionally, their structures were modeled and superposed to show that these domains fold apparently in a similar way in the two proteins, with TM-scores of 0.99 and 0.96 for HMG-box A and B, respectively (Figure 1b).

**Figure 2:**
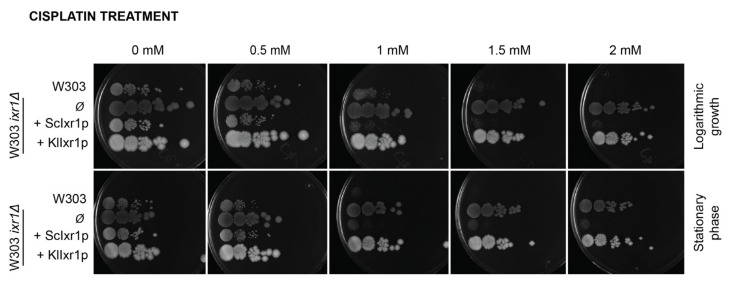
Effect of cisplatin treatment on cell growth (in logarithmic and stationary phase) of strain W303 and derivatives. *ixr1Δ* null strain was transformed with the plasmid pAG426GAL-ccdB, either empty (*Ø*), or containing the *IXR1* ORFs from *S. cerevisiae* or *K. lactis* under the control of the *GAL1* promoter. Serial dilutions of the cells (1, 10^−1^, 10^−2^, 10^−3^, 10^−4^, 10^−5^; from left to right) were made from an initial culture of OD_600_ = 1.0 and were grown at 30 °C for three days on YPGal plates with different concentrations of cisplatin as specified.

The function of the HMG-box domains in DNA binding have been proven in *Sc*Ixr1 [6] and their interaction with DNA has been postulated as a mechanism that, interfering with the machineries of DNA repair, increases cisplatin cytotoxicity [6,27,28]. More recently, we demonstrated that *Sc*Ixr1 participates in the yeast response to cisplatin treatment through the control of ribosome biogenesis by direct binding to promoters of specific genes that regulate rRNA, ribosomal proteins, and RiBi (Ribosone Biogenesis) gene expression, as well as partial overlapping of *Sc*Ixr1 targets and TOR (Target of Rapamycin) signaling pathway components that regulate cell proliferation [29,30]. Since the HMG domains are the only well conserved regions between *Sc*Ixr1 and *Kl*Ixr1, we tested if heterologous expression of *KLLA0E18481g* (*KlIXR1*) in *S. cerevisiae* could complement the increased resistance to cisplatin observed in the *S. cerevisiae ixr1Δ* deletion mutant. The *S. cerevisiae* strains W303 and its isogenic derivative W303-*ixr1Δ* [37] were transformed with the construction YEplac195-*KlIXR1* and cisplatin sensitivity was analyzed in plate by serial dilutions prepared from cultures at logarithmic growth or stationary phase, as described in Materials and Methods. From results obtained (Figure 2), the absence of complementation for this phenotype between *Kl*Ixr1 and *Sc*Ixr1 is concluded in both growth phases.

### 3.2. KlIXR1 Is Not Related to Cisplatin Sensitivity in K. lactis

**Figure 3:**
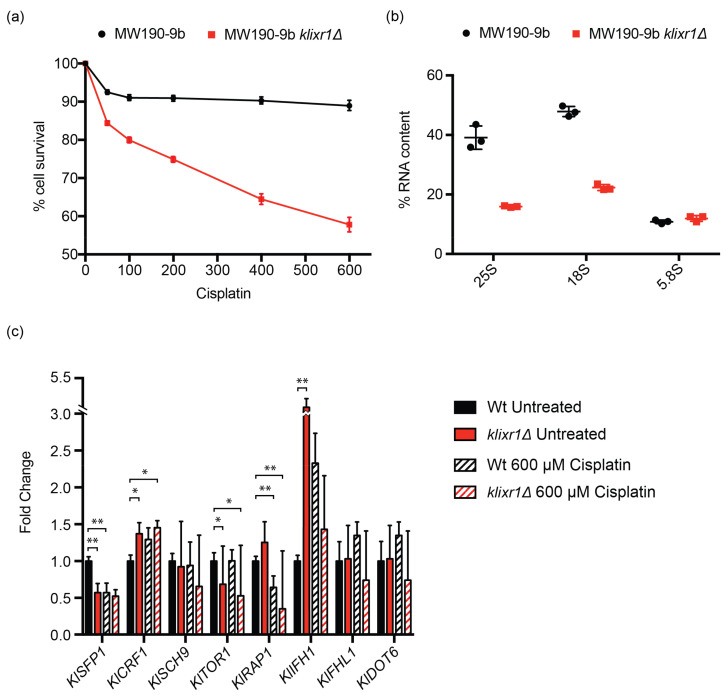
Study of the cellular response of MW190-9b strain (black) and its derivative *ixr1Δ* (red) to cisplatin treatment. (**a**) Resistance to cisplatin in the wild type and *klixr1Δ* null strains. Cells from the *K. lactis* strain MW190-9B and its isogenic derivative MW190-9B-*ixr1Δ* were treated with several concentrations of cisplatin (0, 50, 100, 200, 400, and 600 µM). After 24 h of growth at 30 °C, optical density measurements at 600 nm were taken, and survival rates were calculated by normalization from untreated cultures. (**b**) rRNA cellular content determination by microfluidic-based automated electrophoresis. (**c**) mRNA levels of *KlSFP1*, *KlCRF1*, *KlSCH9*, *KlTOR1*, *KlRAP1*, *KlIFH1*, *KlFHL1,* and *KlDOT6* were analyzed by RT-qPCR, before (solid) and after (streaked) the treatment with 600 µM of the chemotherapeutic agent cisplatin. Housekeeping gene *KlTAF10* was used for gene normalization. * *p* < 0.05; ** *p* < 0.01.

Since *KlIXR1* was unable to complement the function of the *ScIXR1* gene increasing cisplatin citotoxicity, we constructed a *K. lactis ixr1Δ* null strain to test whether the phenotype was intrinsically observable in *K. lactis*. The results show that deletion of *KlIXR1* does not increase cisplatin resistance as reported for the *ScIXR1* gene deletion in *S. cerevisiae* [27], but oppositely, the cytotoxic effect of the drug was increased in the MW190-9b-*ixr1Δ* null mutant (Figure 3a). Cisplatin has a cytotoxic effect upon *K. lactis* cells similar to that caused upon *S. cerevisiae*; however, genes related to this phenotype have not been directly studied in *K. lactis* yet.

### 3.3. KlIxr1 Controls the Expression of Genes Related to rRNA Processing and Ribosome Biogenesis in K. lactis

Considering that we had previously found in *S. cerevisiae* that *Sc*Ixr1 regulates the rRNA processing machinery [24], rRNA cellular content was measured by microfluidics-based automated electrophoresis instruments in the *K. lactis ixr1Δ* null strain and control. Figure 3b shows that *Kl*Ixr1 cellular depletion decreases 25S and 18S rRNA levels, similarly to that previously observed for *S. cerevisiae* [24]. In *S. cerevisiae,* this regulation was mediated by transcriptional *ScSFP1* (Split Finger Protein 1) upregulation and *ScCRF1* (Co-Repressor with FHL1) downregulation [29]. *Sc*Sfp1 is a transcription factor mainly involved in regulation of RiBi genes, but also of ribosomal proteins, by protein translocation from cytoplasm to nucleus after rapid TORC1 kinase activation under growth promoting conditions [47]. Conversely, *Sc*Crf1 is a transcriptional repressor, activated by *Sc*Yak1 (Yet Another Kinase 1) phosphorylation via TOR and PKA pathways during carbon and nitrogen starvation or oxidative stress conditions, to displace the paralogue *Sc*Fhl1 (Fork Head-Like 1) and repress ribosomal protein genes [48]. To analyze regulatory parallelisms in TOR signaling pathways in *S. cerevisiae* and *K. lactis*, mRNA levels of *KlSFP1* (*KLLA0B03047g*) and *KlCRF1* (*KLLA0F13222g*) genes were measured by RT-qPCR in the absence and presence of cisplatin. The results show that *Kl*Ixr1 depletion downregulates the *KlSFP1* gene, meanwhile the repressor *KlCRF1* gene is upregulated (Figure 3c), indicating that *Kl*Ixr1 regulation exerted on ribosome synthesis processes through these two regulators in *K. lactis* is identical to that observed previously in *S. cerevisiae* [29]. 

Additionally, intracellular mRNA levels of another eight genes related to TOR signaling pathway were analyzed by RT-qPCR, including *KlABF1* (*KLLA0F02970g*, previously related to *ScIXR1* regulation [29]), as well as *KlSCH9* (*KLLA0B03586g*), *KlTOR1* (*KLLA0B13948g*), *KlRAP1* (*KLLA0D19294g*), *KlIFH1* (*KLLA0F13222g*), *KlFHL1* (*KLLA0F08206g*), and *KlDOT6* (*KDROE02480*). With the exception of *Sc*Sch9 and *Sc*Tor1 kinases, the other four genes code for transcription factors involved in the regulation of ribosomal protein genes in response to nutritional availability. When nutrients are available, *Sc*Rap1 (Repressor/Activator site binding Protein 1), *Sc*Ifh1 (Interacts with Fork Head 1), and *Sc*Fhl1 bind to ribosomal protein genes and promote active transcriptional rates. Under nutritional stress, *Sc*Abf1 (ARS-Binding Factor 1) is incorporated and *Sc*Ifh1 dissociates to minimize ribosomal protein gene expression and to inhibit cell growth [49]. *Sc*Sch9, another TORC1 direct phosphorylation target that acts through *Sc*Sfp1, is a positive regulator of ribosomal protein and RiBi gene expression [50]. Figure 3c shows that the absence of *Kl*Ixr1 protein reduces the transcriptional levels of *KlTOR1*, but surprisingly *KlIFH1* is highly upregulated, suggesting that different mechanisms have a direct or indirect influence in *K. lactis* regulation of the expression of these genes. 

It was previously found that there is a functional link between the response to cisplatin treatment and regulation of the rRNA processing machinery by *Sc*Ixr1 in *S. cerevisiae* [29]. We also analyzed this possibility in *K. lactis*, but obtained results did not show any significant effect of cisplatin treatment in this regulatory mechanism (Figure 3c).

### 3.4. KlIXR1 Regulates the Expression of the KlHEM13 Gene

**Figure 4:**
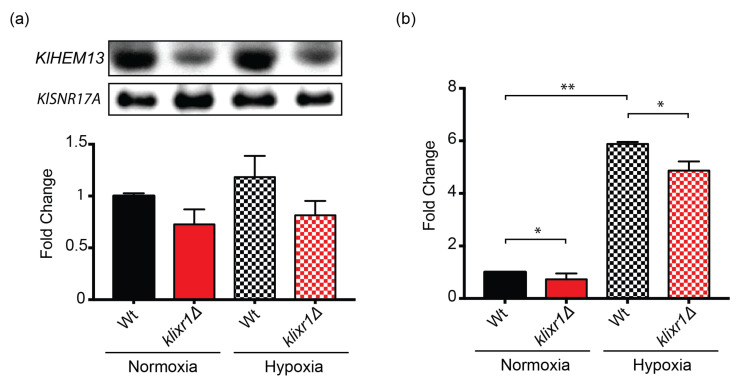
Effect of *KlIXR1* disruption on *KlHEM13* expression under aerobic (solid) or hypoxic conditions (grid) in the MW190-9b strain from *K. lactis* were analyzed by (**a**) Northern blot and (**b**) RT-qPCR experiments. Blots for (**a**) were obtained in triplicate experiments and a representative picture is shown in upper left panel. *KlSNR17A* and *KlTAF10* signal levels were used as references for normalization in (**a**,**b**), respectively. * *p* < 0.05; ** *p* < 0.01.

**Figure 5:**
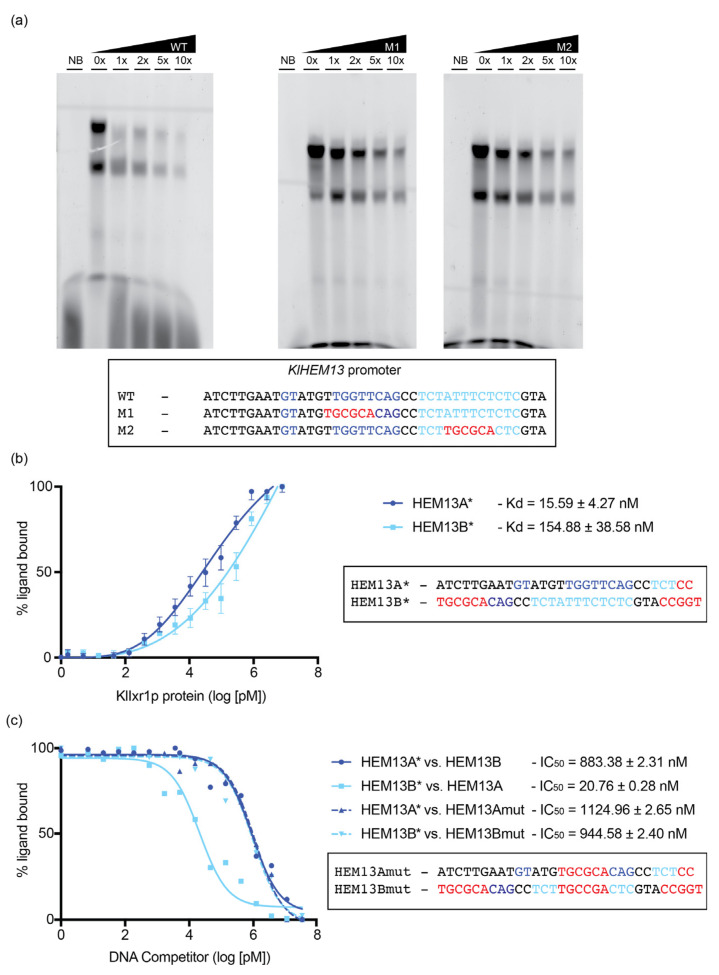
Analysis of *Kl*Ixr1 protein binding to the *KlHEM13* promoter region. (**a**) EMSA assays showing sequence specificity to both binding sites found using the consensus sequences YYYATTGTTCTC and KTTSAAYKGTTYASA previously described [23,24]. Competition assays were conducted with increasing amounts of non-labeled ligands indicated. WT: ligand not mutated; M1: ligand mutated in first site; M2: ligand mutated in second site; NB: no retarded band (no protein added). (**b**) Klotz plots representing quantitative analysis of *Kl*Ixr1 binding to both *KlHEM13* promoter deduced sites. DNA binding measured by fluorescence anisotropy changes of the 5′ fluorescein-labelled DNA (100 nM ligand) upon protein titration (see Section 2). The resulting semi-log binding isotherms were fitted to a 1:1 binding model with non-linear least squares regression. Data points are the average of 3 independent experiments, error bars representing standard deviations. HEM13A: first binding site deduced from KTTSAAYKGTTYASA [23] consensus sequence; HEM13B: second binding site deduced from YYYATTGTTCTC [24] consensus sequence; * indicates FAM labeled ligand. (**c**) Klotz plots representing competition analysis of *Kl*Ixr1 binding to both *KlHEM13* promoter-assayed sites. DNA binding measured by fluorescence anisotropy changes of the 5′ fluorescein-labelled DNA (100 nM ligand) bound to *Kl*Ixr1 protein (200 nM) and upon non-labeled DNA competitor titration (see Section 2). The resulting semi-log binding isotherms were fitted to a logIC50 competitive model with non-linear least squares regression. * indicates FAM labeled ligand.

We have previously described that *Sc*Ixr1 from *S. cerevisiae* controls the aerobic repression and hypoxic activation of genes involved in the adaptation to oxygen levels, including *ScHEM13* [24]. With the aim to elucidate whether a similar regulatory role is functional for *Kl*Ixr1 in *K. lactis*, we analyzed the transcriptional levels of *KlHEM13* (*KLLA0F20075g*) from the *K. lactis* strain MW190-9B and its isogenic derivative MW190-9B-*ixr1Δ*, in normoxic and hypoxic conditions, by Northern blot (Figure 4a) and Real-time qPCR experiments (Figure 4b). The results demonstrated that *Kl*Ixr1 acts as a transcriptional activator in both conditions, independently of oxygen levels. Additionally, we tested the possibility that *Kl*Ixr1 would exert a *cis* regulation on *KlHEM13* promoter. In silico analysis of the *KlHEM13* promoter region, using the AYKGTT core sequence for *Sc*Ixr1 sequence-specific DNA binding, rendering two possible binding sites, both situated very closely between –373 and –352 positions from the translational start site of *KlHEM13*. EMSA assays showed that the *Kl*Ixr1 protein is able to interact to both binding sites, observing two retarded bands, which correlate with stoichiometry 1:1 and 2:1 (protein: ligand) (Figure 5a). To calculate equilibrium dissociation constants, both putative binding sites were analyzed by fluorescence anisotropy. The results showed that binding affinity of *Kl*Ixr1 for the first site (ATCTTGAATGTATGTTGGTTCAGCCTCTCC) is in the low nanomolar range, meanwhile the binding affinity for the second site (TGGCCCAGCCTCTATTTCTCTCGTACCGGT) is ten times lower (Figure 5b,c). Additionally, mutations of the putative core sequences combined with EMSA or FA assays indicate that *Kl*Ixr1 protein binds to both sites in a sequence-specific manner (Figure 5a,c).

### 3.5. KlIxr1 Is Implicated in the Response to Hydrogen Peroxide and Cadmium-Metals

**Figure 6:**
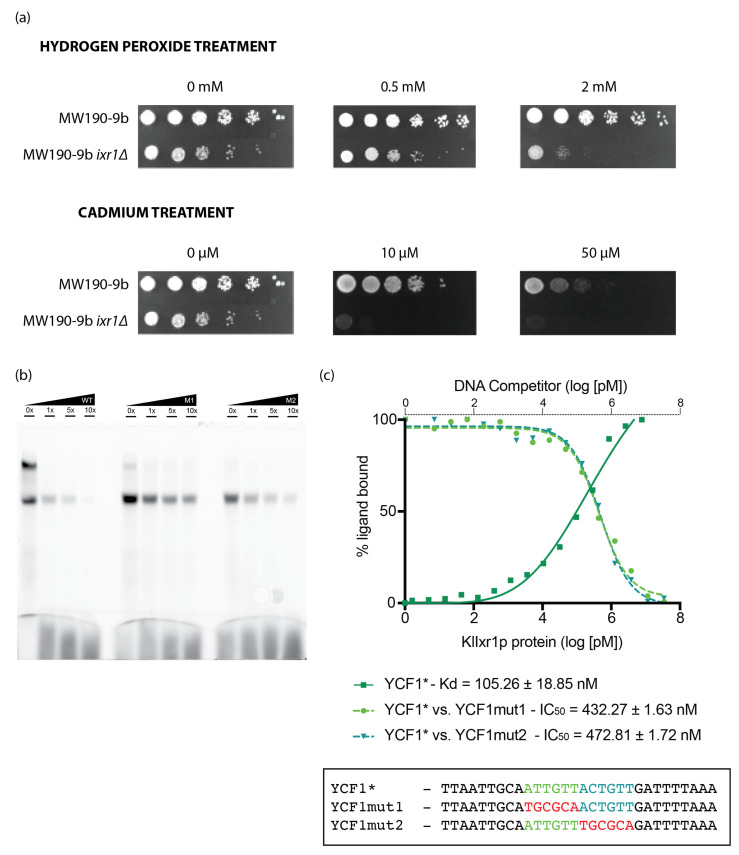
Analysis of the cellular response of MW190-9b strain and its derivative *ixr1Δ* to hydrogen peroxide and cadmium treatments. (**a**) Resistance to H_2_O_2_ and cadmium in the wild type and *klixr1Δ* null strains. Serial dilutions of the cells (1, 10^−1^, 10^−2^, 10^−3^, 10^−4^, 10^−5^; from left to right) were made from an initial culture of OD_600_ = 1.0 and were grown at 30 °C for three days on CM plates supplemented with the indicated concentrations of compound. (**b**) EMSA assays for the *KlYCF1* promoter region contains two putative contiguous binding sites for *Kl*IXR1 deduced by using the AYKGTT core consensus sequence [23] in silico searches. Competition assays were conducted with increasing amounts of non-labeled ligands indicated. WT: ligand not mutated; M1: ligand mutated in first site; M2: ligand mutated in second site. (**c**) Klotz plots representing quantitative analysis of *Kl*Ixr1 binding to *KlYCF1* promoter-deduced site. DNA binding measured by fluorescence anisotropy changes of the 5′ fluorescein labelled DNA (100 nM ligand) upon protein titration (Section 2). The resulting semi-log binding isotherms were fitted to a 1:1 binding model with non-linear least squares regression. Data points are the average of 3 independent experiments. Competition analysis of *Kl*Ixr1 binding to both *KlYCF1* promoter deduced sites were measured by fluorescence anisotropy changes of the 5′ fluorescein labelled DNA (100 nM ligand non mutated) bound to *Kl*Ixr1 protein (200 nM) and upon non-labeled DNA competitor titration (*Kl*Ixr1 binding sites individually mutated). The resulting semi-log binding isotherms were fitted to a logIC50 competitive model with non-linear least squares regression. * indicates FAM labeled ligand.

We had previously demonstrated the implication of *Sc*Ixr1 in the cellular response to hypoxia [24] and oxidative stress, two conditions tightly connected in *S. cerevisiae* [51]. With the aim to determine regulatory parallelisms in *K. lactis*, the wild-type strain MW190-9b and its derivative *ixr1Δ* null mutant were grown in the presence of increasing concentrations of hydrogen peroxide. Figure 6a shows that the strain MW190-9b-*ixr1Δ* was less resistant to oxidative stress caused by 0.5 or 2 mM H_2_O_2_, indicating that *Kl*Ixr1 is also important for conferring resistance to such stress. Additionally, and since oxidative stress can be also induced by high concentrations of metals [52], we tested the effect of cadmium. A sensitive phenotype was found in the *ixr1Δ* null mutant to the presence of 10 and 50 µM of cadmium (Figure 6a). Yeast Cadmium Factor 1 (*Sc*YCF1) is one of the main cellular elements to confer cadmium resistance in *S. cerevisiae* through a mechanism causing vacuolar sequestration of GSH-conjugated cadmium, bis (glutathionate) cadmium (Cd-GS_2_) [53,54,55]. It was reported that *Kl*Rox1 regulates the expression of *KlYCF1* gene and mediates the response to cadmium metals [35]. The analysis of the *KlYCF1* promoter region (*KLLA0F20075g*) by RSA tools provided two consecutive putative *Kl*Ixr1 binding regions at -568 and -562 positions from the coding start codon. In order to analyze the relationship between cadmium resistance associated to *KlIXR1* gene expression and a possible *cis* regulatory role of *Kl*Ixr1 protein on *KlYCF1* promoter, EMSA assays were performed. Figure 6b shows that *Kl*Ixr1 binds to *KlYCF1* promoter sequences containing both putative sites. These binding evidences lower affinity (K_d_ = 105.26 ± 18.85 nM) in comparison with binding to the *KlHEM13* promoter region (with K_d_ = 15.59 ± 4.27 nM for HEM13A and K_d_ = 154.88 ± 38.58 nM for HEM13B) (Figure 5 and Figure 6).

### 3.6. Deletion of KlIXR1 Causes Petite Colonies That Are Not Related to Carbon Source, Cell Cycle Control, or Defects in Heme Production

**Figure 7:**
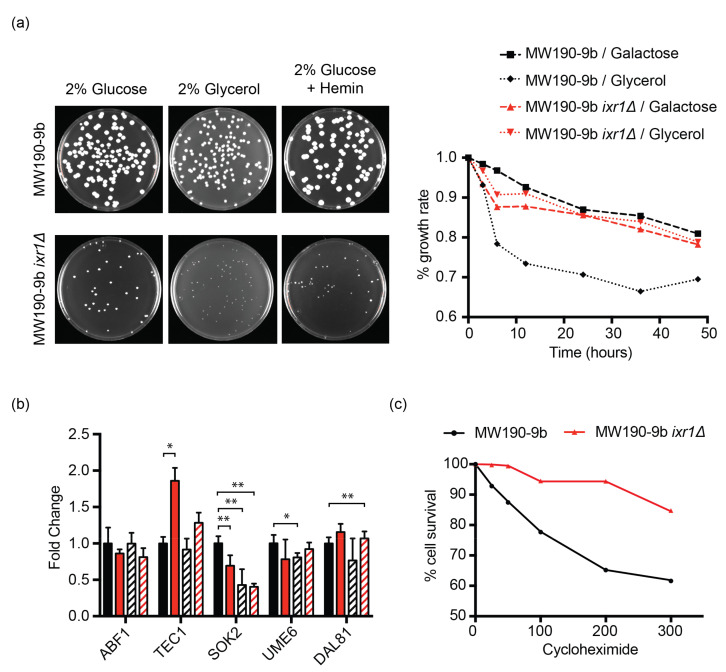
Analysis of *petite* phenotype in MW190-9b *ixr1Δ* null mutant. (**a**) (left) Colony size of MW190-9b strain and its derivative *ixr1Δ* in 2% *w/v* glucose, 2% *w/v* glycerol, or 2% *w/v* glucose supplemented with 50 µg/mL hemin. (right) Growth tracking of MW190-9b strain (black) and its derivative *ixr1Δ* (red) 2% *w/v* galactose or 2% *w/v* glycerol during 48h. Growth rates were calculated using respective 2% *w/v* glucose cultures as normalization elements. (**b**) RT-qPCR results of *KlABF1*, *KlTEC1*, *KlSOK2*, *KlUME6,* and *KlDAL81* genes in wild-type (black) and *ixr1Δ* null strain (red), before (solid) and after (streaked) the treatment with 600 µM of the chemotherapeutic agent cisplatin. Housekeeping gene *KlTAF10* was used for gene normalization. * *p* < 0.05; ** *p* <0.01. (**c**) Cellular response to cycloheximide treatment. Cell cultures of the *K. lactis* strain MW190-9B (black) and its isogenic derivative MW190-9B-*ixr1Δ* (red) were treated with several concentrations of cycloheximide (0, 25, 50, 100, 200, and 300 µg/mL). After 24 h of growth at 30 °C, optical density measurements at 600 nm were taken, and survival rates were calculated by normalization from untreated cultures.

A phenotype observed in the *K. lactis ixr1Δ* null strain and not previously reported for the *S. cerevisiae ixr1Δ* null strains is the smaller size of the colonies (*petites*). We further investigated this phenotype by diverse approaches. We first verified if the phenotype was dependent on carbon source and we found that it is produced both in fermentable (glucose) and non-fermentable (glycerol) carbon sources (Figure 7a). The observation of *petite* growth in *K. lactis* also in glucose could be attributable to a minor fermentative capacity in *K. lactis* than in *S. cerevisiae*. Therefore, the growth defect caused by *Kl*Ixr1 depletion is not compensated by fermentative metabolism. Additionally, and considering that *Kl*Ixr1 activates the transcription of the *KlHEM13* gene as above reported, we also tested if this phenotype could be attributed to heme deficiency/shortness. To test this hypothesis, we plate the strain in media supplemented with hemin and results show that externally provided heme-precursor does not overcome this growth decrease (Figure 7a).

Transcriptomic data revealed *Sc*Ixr1 is a master regulator of regulators related to cell cycle progression and cell growth [29], since *Sc*Ixr1 function modulates mRNA levels of several key transcriptional factors associated to these processes, including *ScTEC1* (Transposon Enhancement Control 1), *ScSOK2* (Suppressor Of Kinase 2), *ScUME6* (Unscheduled Meiotic gene Expression 6) (*KDROA03340*), or *ScDAL81* (Degradation of Allantoin 81) (*KDROE03280*) among others [29]. *Sc*Tec1 participates in the regulation of several signaling pathways, which respond to nutrient availability in a TOR1C-dependent manner, and control cellular developmental programs [56]. On the other hand, *Sc*Sok2 interacts with the *Sc*Tup1-*Sc*Cyc8 co-repressor complex to downregulate the expression of certain genes under plenty nutrient availability [57,58,59]. In *K. lactis,* deletion of *KlIXR1* decreases significantly the mRNA levels of *KlSOK2* (*KLLA0F04840g*), meanwhile *KlTEC1* (*KLLA0E12497g*) was upregulated (Figure 7b). More surprisingly, *Kl*Ixr1 depletion causes a resistant phenotype in presence of the protein translation inhibitor cycloheximide (Figure 7c), which blocks meiosis by cell cycle arrest at G1 phase.

### 3.7. KlIxr1 and the Regulation of Genes Related to De Novo dNTP Synthesis

In *S. cerevisiae*, during the normal cell cycle, the adequate supply of dNTPs, which are necessary for DNA synthesis, is regulated through modulation of the activity of ribonucleotide reductase (RNR) or the expression of the RNR genes [60]. The genes *ScRNR1* and *ScRNR3* encode the large subunit of RNR and *ScRNR2* and *ScRNR4* encode the small subunit. Transcriptional regulation of *ScRNR3*, *ScRNR2,* and *ScRNR4* is repressed by the transcriptional factor *Sc*Crt1 (Regulatory Factor X 1) and its phosphorylation, which is *Sc*Mec1-*Sc*Rad53-*Sc*Dun1–dependent after DNA damage or replication stress, promotes activation of their transcription [61]. The kinase activity of *Sc*Dun1 (DNA-damage Uninducible 1) also targets for degradation *Sc*Sml1 (Suppressor of Mec1 Lethality 1), a protein inhibitor of RNR, and *Sc*Dif1 (Damage-regulated Import Facilitator 1), a protein that regulates the nuclear retention of *Sc*Rnr2 and *Sc*Rnr4, thus promoting RNR activity [62]. *Sc*Rox1 and *Sc*Mot3 participates in *ScRNR3*, *ScRNR2,* and *ScRNR4* repression [63]. The transcription of *ScRNR1* is independent of *Sc*Crt1, *Sc*Rox1, and *Sc*Mot3. The deletion of *ScIXR1* results in decreased dNTP levels due to a reduced *ScRNR1* expression and to *Sc*Ixr1 binding to the *ScRNR1* promoter. The control of *ScRNR1* by *Sc*Ixr1 is *Sc*Mec1-*Sc*Rad53 dependent, but independent of *Sc*Dun1 [31]. 

The biosynthesis of dNTPs has not been studied in *K. lactis*, but exploring the sequence of the *K. lactis* genome we have found similarities and differences in the putative homologs of the genes related to the control of the dNTPs pool in comparison with *S. cerevisiae*. In *K. lactis* there are two ORFs (*KLLA0C07887g* and *KLLA0F01188g*) encoding proteins with significant homology to *S. cerevisiae Sc*Rnr1 and *Sc*Rnr3, the large subunits of RNR. It is not possible to establish a unique correspondence based merely in their sequence similarity, since *KLLA0C07887g* is more similar to both *Sc*Rnr1 and *Sc*Rnr3 than *KLLA0F01188g*. Regarding the small subunit of RNR in *K. lactis,* there is only one gene encoding this subunit (*KLLA0F15103g*). The machinery of signal transduction after DNA-damage or replicative stress (*Sc*Rad53, *Sc*Mec1 and *Sc*Dun1) is well conserved between both yeast species. However, there is low conservation of the transcriptional regulators *Sc*Crt1, *Sc*Crt10 [64] (absent) or other regulatory factors affecting RNR activity like *Sc*Sml1 or *Sc*Dif1. 

**Figure 8:**
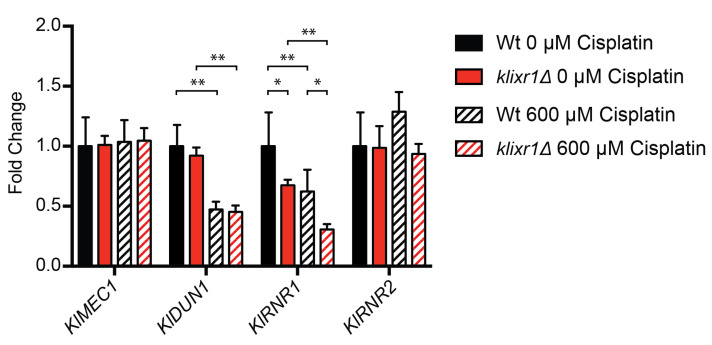
Regulatory role of *Kl*Ixr1 in the *Kl*Mec1-*Kl*Rad53-*Kl*Dun1–dependent ribonucleotide reductase pathway of *K. lactis*. mRNA levels of *KlMEC1*, *KlDUN1*, *KlRNR1,* and *KlRNR2* were analyzed by RT-qPCR in the MW190-9b strain (black) and its derivative *ixr1Δ* (red), before (solid) and after (streaked) the treatment with 600 µM of the chemotherapeutic agent cisplatin. Housekeeping gene *KlTAF10* was used for gene normalization. * *p* < 0.05; ** *p* < 0.01.

RT-qPCR experiments were carried out in order to assess the role of *Kl*Ixr1 in the regulation and maintenance of dNTPs pools by analyzing the transcriptional levels of the regulators *KlMEC1* (*KLLA0C15785g*) and *KlDUN1* (*KLLA0E01585g*) and the ribonucleotide reductase genes *KlRNR1* and *KlRNR2*. The results showed that there exists a clear parallelism between transcriptional regulation of this pathway in *S. cerevisiae* and *K. lactis*. In this sense, *KlRNR1* is downregulated in the *K.lactis ixr1Δ* strain. However, *KlRNR2* and the key regulators *KlMEC1* and *KlDUN1* (upstream of *ScIXR1* in the regulatory pathway of *S. cerevisiae*) are not affected by *KlIXR1* deletion (Figure 8). 

Additionally, we have analyzed transcript levels of these genes in the presence of 600 µM cisplatin to induce DNA lesions. The results obtained showed that the expression *KlRNR1* and *KIRNR2* is increased in the wild-type, meanwhile the reduction of *KlRNR1* transcript levels in the *Klixr1Δ* null strain are even more abrupt (Figure 8), as previously described for *S. cerevisiae* in the presence of 4-nitroquinoline 1-oxide (4-NQO) or hydroxyurea (HU) [31].

## 4. Discussion

Nowadays, eukaryotic unicellular models are useful for the easy study of orphan genes without assigned functions and for massive screening of drug targets. Most studies about the hypoxic and oxidative stress responses in eukaryotes, and their connections, were developed initially on *S. cerevisiae* cells with a predominant fermentative metabolism [34]. *S. cerevisiae* mutants have been widely used as research models in aging [65] or in human pathologies [66]. Frequently, the mechanisms discovered with this yeast proved to be conserved in multicellular eukaryotes. However, human cells, including those from the nervous system, have usually a respiratory metabolism, differing from *S. cerevisiae* cells. In this sense, *K. lactis* arises as an alternative eukaryote model more suitable for studies about the nervous system or their pathologies, since this yeast has a predominantly respiratory metabolism, although sharing essential regulatory machineries with *S. cerevisiae* [67]. Also important for the selection of *K. lactis* as an eukaryotic model is the fact that it is evolutionary derived from an ancestral yeast previous to the whole-genome duplication (WGD) that took place over 100 million years ago and resulted later, after genomic losses and chromosome reorganizations, in the existence of two copies of some genes in *S. cerevisiae* [68]. Each paralog likely sub-functionalized to carry out some, but not all, of its previous functions so that, a more complex functional network arose [69]. 

HMGB proteins are not extensively studied in *K. lactis*, although their transcriptional regulatory function might predictably influence the respiratory metabolism of this yeast. In the present work, we show evidence of the cellular functions of the HMGB protein *Kl*Ixr1, finding parallelisms with *Sc*Ixr1 functions controlling the expression of genes that are modulators of oxidative metabolism and oxidative stress response, dNTP cellular levels, and ribosome biogenesis.

In *S. cerevisiae*, one of the principal sensors in the yeast response to changes in oxygen levels is heme, since its enzymatic synthesis is oxygen-dependent. The heme biosynthetic pathway is well conserved between *S. cerevisiae* and *K. lactis*. Both yeasts have eight genes encoding the enzymes for the biosynthesis of heme, and up to three (*KlHEM1*, *KlHEM12,* and *KlHEM13*) share functional equivalence confirmed experimentally by cross-complementation [70,71,72]. Although *K. lactis* homologs of key regulators (*Sc*Hap1 and *Sc*Rox1) of the biosynthesis of heme in *S. cerevisiae* have been characterized, their sequence and function diverge notably from those described in *S. cerevisiae* [35,73]. Here, we show that transcriptional activation of *KlHEM13* is also dependent on the HMGB protein *Kl*Ixr1p, as occurred with *Sc*Ixr1 in *S. cerevisiae* [24]. Moreover, both proteins share similar DNA binding sequences in their target promoters, as shown by EMSA and fluorescence anisotropy experiments. 

Several *S. cerevisiae* genes that are induced during hypoxia are also related to the oxidative stress response caused by hydrogen peroxide or metals, including *ScCUP1* (CUPrum 1), *ScCUP2* (CUPrum 1), *ScHSP12* (Heat Shock Protein 12), *ScFMP46* (Found in Mitochondrial Proteome 46), *ScGRE1* (stress Responsive Gene 1), *ScALK1*, *ScMGM1* (Mitochondrial Genome Maintenance 1), or *ScSOD1* (SuperOxide Dismutase 1), among others [51,65,74]. In this sense, we confirmed that depletion of *Kl*Ixr1 protein in *K. lactis* also produces an increased sensitivity to the presence of hydrogen peroxide and cadmium metal, as occurred in *S. cerevisiae* [23,75]. Furthermore, we show for the first time that *Kl*Ixr1 is implied in the transcriptional regulation of the *KlYCF1* gene, involved in cadmium detoxification, and binds to its promoter. Interestingly, other regulators of the respiratory metabolism in response to changes of oxygen levels, *Kl*Hap1 and *Kl*Rox1, have been previously related to the oxidative stress response in *K. lactis* [30,37]. 

It was previously described that the role of *Sc*Ixr1 is as a “regulator of regulators” directly or indirectly controlling the expression of a set of 33 yeast transcriptional factors mostly involved in the regulation of cell growth and cell cycle progression. These transcriptional regulators are downregulated in the *Scixr1Δ* mutant strain [29]. In *S. cerevisiae*, these regulatory networks sense nutrient availability, external stimuli, or DNA damage through TOR complexes known as Tor Complex 1 (*ScTORC1*, TOR complex 1) and Tor Complex 2 (*ScTORC2*, TOR complex 2) [76]. *ScTORC1* is sensitive to rapamycin and controls protein synthesis, mRNA synthesis and degradation, ribosome biogenesis, and autophagy. Rapamycin is currently used clinically as an immunosuppressive drug for organ and tissue transplant recipients and a chemotherapy agent against a variety of solid cancers because of its antiproliferative properties [77]. *S. cerevisiae* cells treated with rapamycin display phenotypes associated with nutrient depletion including G1 cell cycle arrest, cellular volume expansion, protein synthesis inhibition, glycogen accumulation, and autophagy [78]. In *K. lactis*, although only a TOR complex exists, we have found that *Kl*Ixr1 is also implied in the control of ribosomal production and cellular growth by regulation of rRNA levels, as well as transcription of several RiBi genes and other transcriptional factors that modulate ribosomal protein gene expression, including *KlSFP1*, *KlCRF1*, *KlTOR1,* or *KlIFH1*.

Cellular division depends on the maintenance of dNTP pools for DNA synthesis and genes encoding ribonucleotide reductases (RNR) are tightly regulated [26]. We have also shown that *Kl*Ixr1 modulates transcriptional activation of the *KlRNR1* gene, a mechanism previously described in *S. cerevisiae* [31]. RNR expression is tightly controlled by the genome integrity checkpoint, a conserved signaling pathway that is regulated in *S. cerevisiae* by the *Sc*Mec1 (homologous to human ATR) and *Sc*Rad53 (homologous to human Chk1) kinases. Under DNA damage or DNA replication stress, this pathway promotes the activation of *ScRNR2*, *ScRNR3,* and *ScRNR4* through the *Sc*Mec1-*Sc*Rad53-*Sc*Dun1 kinase cascade, which targets and inactivates the transcriptional inhibitor *Sc*Crt1 [62]. However, upregulation of *Sc*RNR1 in response to DNA damage requires *Sc*Mec1 and *Sc*Rad53, but not *Sc*Dun1. If this molecular mechanism is also maintained, *K. lactis* will need to be elucidated in future experiments.

It is interesting to note that despite all similitudes found between *Kl*Ixr1 and *Sc*Ixr1, showing that *Kl*Ixr1 is also a master regulator of oxidative metabolism, oxidative stress response, cell growth, and cell division in *K. latis*, several functions are not conserved between *Sc*Ixr1 and *Kl*IXr1. We have found that *Kl*Ixr1p is not able to supplement the absence of *Sc*Ixr1 in *S. cerevisiae* and unable to revert the phenotype of increased resistance to cisplatin treatment associated to the null mutation [37]. Furthermore, *KlIXR1* gene knockout generates the opposite phenotype in *K. lactis*, conferring a sharp sensitivity to cisplatin treatment. Some transcriptional regulators that were previously related to cisplatin resistance or ribosomal biosynthesis in *S. cerevisiae* [73,74,75] are also differently regulated by *Sc*Ixr1 and *Kl*Ixr1 in both species, respectively. We have observed these differences comparing mRNA changes of *KlABF1*, *KlUME6,* or *KlDAL81* after *Kl*Ixr1 depletion with those previously reported in *S. cerevisiae* after *Sc*IXr1 depletion [29]. Related to this, we have found that *Kl*Ixr1 depletion causes a resistant phenotype to cyclohexymide not previously described for the *Scixr1∆* null mutant, and that could be associated to alterations of certain ribosomal protein targets of the drug [76], among other causes. Future structural studies are necessary to dilucidate if non-conserved domains in the Ixr1 proteins from *S. cerevisiae* and *K. lactis* are responsible of differences observed in their functions in reference to responses to cisplatin or cyclohexymide treatments.

## 5. Conclusions

Taken together with the data reported here, Ixr1 from *K. lactis* shows similarities with its *S. cerevisiae* counterpart to how these two proteins participate in the transcriptional regulation of genes related to cellular growth, dNTP levels, and ribosome biosynthesis, as well as of genes implicated in the cellular response changes in oxygen availability, which consequently alters cellular levels of heme and ROS. This supports the idea that these regulation mechanisms are essentially well conserved for Ixr1 in *S. cerevisiae* and *K. lactis* yeasts, which differ in lifestyle with respect to carbon sources. However, the finding of new roles for *Kl*Ixr1 protein in the response to platinum compounds and the occurrence of a petite phenotype after *Kl*IXR1 gene deletion reveal that it is necessary to further understanding of the mechanisms of *Kl*Ixr1 function and also a broader knowledge of the regulatory pathways associated to all these processes in *K. lactis*.

## Data Availability

Not applicable.

## Supplementary Materials

The following are available online at https://www.mdpi.com/article/10.3390/biom11091392/s1, Table S1: Oligonucleotides used in this study; Figure S1: Verification of MW190-9b *klixr1Δ* null strain by PCR and agarose gel electrophoresis; Figure S2: SDS-PAGE gel of *Kl*Ixr1 purification.
