# Supplementary material for: The HMGB Protein KlIxr1, a DNA Binding Regulator of Kluyveromyces lactis Gene Expression Involved in Oxidative Metabolism, Growth, and dNTP Synthesis
Source: Biomolecules. 2021 Sep 21;11(9):1392. doi: 10.3390/biom11091392 (PMC8465852; doi:10.3390/biom11091392)
Supplement: Supplementary file 1 [file biomolecules-11-01392-s001.zip › biomolecules-1361315-supplementary.pdf]

Supplementary data

Table S1. Oligonucleotides used in this study. Lower case indicates a sequence added for cloning purposes. Bold indicates a mutated sequence. Asterisk indicates fluorescein-labeled (FAM) oligonucleotides. Numbering is considering +1 for the A in the first ATG codon. W, Watson; C, Crick.

Name	Sequence	Gene	Strand (W/C)	Added site	Hybridization position
**IXR1 knockout in MW190-9b**					
ECV719	GGGgcatgcCAGACGGGCCAGACAGC	*KlIXR1*	W	*SphI*	-698
ECV720	GGGgtcgacCGGAATGTACGTTGGATCTGC	*KlIXR1*	C	*SalI*	+2005
ECV763	TTTgcggccgcTGTTCGCTATGTCCTTGGTT	*KlIXR1*	C	*NotI*	+12
ECV764	TTTgcggccgcGCTGAAGCTGCTGCAG	*KlIXR1*	W	*NotI*	+1374
ECV765	GTCTAGCACTCTCCCAGTG	*KlIXR1*	W	-	-640
ECV766	GAGAAGTCGTATATGCCTTCATTAC	*KlIXR1*	C	-	+1968
ECV315K2	CGCCTTAATTAACCCGGGGAT	*KanMX4*	W	-	+1322
ECV314K3	GCCTCGACATCATCTGCCCAG	*KanMX4*	C	-	+45
ECV808AR	TTAGGAAACCATTTGTACATAGATTTGTG	*KlIXR1*	W	-	-822
ECV809AR	TCCTATTCCACCTTTCAGGATACAAC	*KlIXR1*	C	-	+2241
**Northern blot**					
ECV051	GGGGTCGACTTGGCAGAAGAACGAATAACAGA	*KlHEM13*	W		+29
ECV053	GGGGGATCCCATCGAGGCCTTGAGGAAAA	*KlHEM13*	C		-764
U3F	CGACGTACTTCAGTATGTAA	*KlSNR17A*	W		+3
U3R	ATTTGTACCCACCCATAGAG	*KlSNR17A*	C		+475
**RT-qPCR**					
AVV83q	AAATGGAAGACAACCCGCC	*KlTAF10*	W	-	+217
AVV84q	TTACGAATTTCTGTGTAGCAAGAGC	*KlTAF10*	C	-	+315
AVV85q	ACCTGGAATGCGGACGG	*KlHEM13*	W	-	+558
AVV86q	TGTCCAAAGCATCCTTGTGC	*KlHEM13*	C	-	+662
AJVV164f	GGAATGTTGGTGGTGGTAAGA	*KlSFP1*	W	-	+374
AJVV165r	TGGCCACAGCAGCTATAATC	*KlSFP1*	C	-	+497
AJVV166f	GAGTTACCCAAGCCCAGAAA	*KlABF1*	W	-	+421

AJVV167r	CAGAGGAAGATCTTGGCCTAAC	*KlABF1*	C	-	+566
AJVV168f	GCCACTATCTTCGGGAGTAAAT	*KlTEC1*	W	-	+1452
AJVV169r	CAGTGTTCATGGTTGCAGTTC	*KlTEC1*	C	-	+1555
AJVV170f	CCTCAAGTAGCACAGGTTTCTC	*KlSOK2*	W	-	+199
AJVV171r	CGCATAGCCCAGATACTGATTT	*KlSOK2*	C	-	+324
AJVV172f	GCGACAAAGCGGACATAGA	*KlUME6*	W	-	+761
AJVV173r	CAAGGATCGGCGAAGATGAA	*KlUME6*	C	-	+895
AJVV174f	CGTTAATGGTGGTGACTCTCTC	*KlDAL81*	W	-	+21
AJVV175r	CAACTGAAGTGGTCTCCTTACC	*KlDAL81*	C	-	+139
AJVV176f	AGATATTTCACCCACGCTTCTC	*KlCRF1*	W	-	+1021
AJVV177r	ACCTTCGATGGAATCCTCTTTC	*KlCRF1*	C	-	+1144
AJVV178f	CCTGTTCCTGAGGCTAGAAATG	*KlRNR1*	W	-	+1534
AJVV179r	TTCAAATGGAGTACGGCAGAG	*KlRNR1*	C	-	+1635
AJVV180f	GATACTTCATGGATGCTCTACCC	*KlRNR2*	W	-	+926
AJVV181r	TACCCAAGGCGACCAATAATC	*KlRNR2*	C	-	+1030
AJVV182f	CTGGGATGACGACGAATTAGAT	*KlDUN1*	W		+1164
AJVV183r	ACGTAAGAAGGTGTTCCACATAG	*KlDUN1*	C	-	+1274
AJVV184f	GACTCGCTCAAGATGGCTTAC	*KLMEC1*	W	-	+3751
AJVV185r	TTCCGTTGGCTGCTTGATAG	*KLMEC1*	C	-	+3850
AJVV218f	GATTGCGTTCGGTAAAGTCAAG	*KlSCH9*	W	-	+1608
AJVV219r	GGCACCAAGTCTATGTTTAGGA	*KlSCH9*	C	-	+1713
AJVV220f	CAGATCCAGTTGCCGAGATTAG	*KlTOR1*	W	-	+1901
AJVV221r	GACTTCGTCATTCAGAGCCATAA	*KlTOR1*	C	-	+2043
AJVV222f	CTGAGTCTTCCGGGTTTGTT	*KlRAP1*	W	-	+320
AJVV223r	CGTTGATCCCTGTTGCTATCT	*KlRAP1*	C	-	+451
AJVV224f	GAAGGAGGTACAATGGCAGTAG	*KlIFH1*	W	-	+370
AJVV225r	GTACTCGAACTGTCGCTATCATC	*KlIFH1*	C	-	+476
AJVV226f	AACACGCCTGCCTCTAATAC	*KlFHL1*	W	-	+687
AJVV227r	CGATCGCCTTCCGATAATAACT	*KlFHL1*	C	-	+819
AJVV228f	CCCATTCCTTGAGAGACCATTC	*KlDOT6*	W	-	+1511
AJVV229r	GTTCATTAGCAGCCTGTGTTTG	*KlDOT6*	C	-	+1630
***Kl*Ixr1 protein production**					
AVV021	GCGGattaatATGAATCTCAATAACCAA GGACATAGC	*KlIXR1*	W	*VspI*	+1
AVV022	GGGaagcttTCATTCTGCAGCAGCTTCA GC	*KlIXR1*	C	*HindIII*	+1374
AJVV024f	GGGGacaagtttgtacaaaaaagcaggctTCATG**C** **ACCATCACCATCACCAT**AATCTCAAT	*KlIXR1*	W	*attB1*	+4

	AACCAAGGACATAGCGAACATCAGG				
AJVV025r	GGGGaccactttgtacaagaaagctgggtCCTATT CTGCAGCAGCTTCAGCTTCATGTTCCG	*KlIXR1*	C	*attB2*	+1374
**EMSA & FA assays**					
AJVV009f*	ATCTTGAATGTATGTTGGTTCAGCCTC TATTTCTCTCGTA	*KlHEM13*	W	-	-389
AJVV010r	TACGAGAGAAATAGAGGCTGAACCA ACATACATTCAAGAT	*KlHEM13*	C	-	-389
AJVV011f	ATCTTGAATGTATGTGCGCACAGCCT CTATTTCTCTCGTA	*KlHEM13*	W	-	-389
AJVV012r	TACGAGAGAAATAGAGGCTG***TGCGC*** ***A***CATACATTCAAGAT	*KlHEM13*	C	-	-389
AJVV013f	ATCTTGAATGTATGTTGGTTCAGCCTC TTGCGCACTCGTA	*KlHEM13*	W	-	-389
AJVV014r	TACGAG***TGCGCA***AGAGGCTGAACCA ACATACATTCAAGAT	*KlHEM13*	C	-	-389
AJVV015f	ATCTTGAATGTATGTTGGTTCAGCCTC TATTTCTCTCGTA	*KlHEM13*	W	-	-389
AJVV029f*	TTAATTGCAATTGTTACTGTTGATTTT AAA	*KlYCF1*	C	-	-578
AJVV030f	TTAATTGCAATTGTTACTGTTGATTTT AAA	*KlYCF1*	W	-	-578
AJVV031r	TTTAAAATCAACAGTAACAATTGCAA TTAA	*KlYCF1*	C	-	-578
AJVV045f*	ATCTTGAATGTATGTTGGTTCAGCCTC **TCC**	*KlHEM13*	W	-	-389
AJVV046r	**GG**AGAGGCTGAACCAACATACATTC AAGAT	*KlHEM13*	C	-	-389
AJVV047f	ATCTTGAATGTATGTTGGTTCAGCCTC **TCC**	*KlHEM13*	W	-	-389
AJVV048f	ATCTTGAATGTATG***TGCGCA***CAGCCT CT**CC**	*KlHEM13*	W	-	-389
AJVV049r	**GG**AGAGGCTG***TGCGCA***CATACATTCA AGAT	*KlHEM13*	C	-	-389
AJVV050f*	**TGGCC**CAGCCTCTATTTCTCTCGTA**CC** **GGT**	*KlHEM13*	W	-	-369
AJVV051r	**ACCGG**TACGAGAGAAATAGAGGCTG **GGCCA**	*KlHEM13*	C	-	-369
AJVV052f	**TGGCC**CAGCCTCTATTTCTCTCGTA**CC** **GGT**	*KlHEM13*	W	-	-369
AJVV053f	**TGGCC**CAGCCTCT***TGCGCA***CTCGTA**C** **CGGT**	*KlHEM13*	W	-	-369

AJVV054r	**ACCGG**TACGAG***TGCGCA***AGAGGCTG **GGCCA**	*KlHEM13*	C	-	-369
AJVV055f	TTAATTGCA**TGCGCAACTGTT**GATTT TAAA	*KlYCF1*	W	-	-578
AJVV056r	TTTAAAATC**AACAGTTGCGCA**TGCAA TTAA	*KlYCF1*	C	-	-578
AJVV057f	TTAATTGCA**ATTGTTTGCGCA**GATTT TAAA	*KlYCF1*	W	-	-578
AJVV058r	TTTAAAATC**TGCGCAAACAAT**TGCAA TTAA	*KlYCF1*	C	-	-578

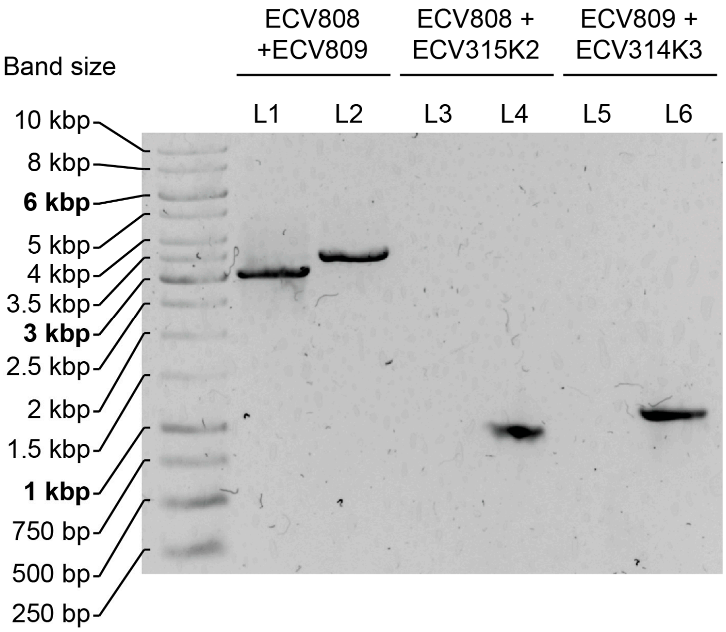

Figure S1: Verification of MW190-9b-*klixr1Δ* null strain by PCR and agarose gel electrophoresis. Genomic DNA from the wild type (lanes L1, L3 and L5) or the derivative with the *KlIXR1* gene knocked out by the insertion of the cassette with the kanMX4 resistance gene (lanes L2, L4 and L6) were used as templates. Expected band size: L1: 3088 bp; L2: 3245 bp; L3: no band; L4: 953 bp; L5: no band; L6: 1035 bp.

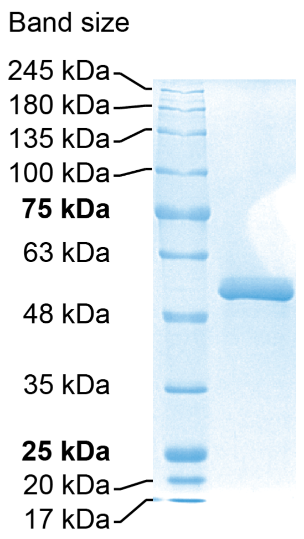

Figure S2: SDS-PAGE gel of *Kl*Ixr1 protein. The protein was produced by heterologous expression in *Escherichia coli* and fully isolated up to a high grade of purity. Expected molecular weight: 51.3 kDa.
